# Analysis and verification of *N*^6^-methyladenosine-modified genes as novel biomarkers for clear cell renal cell carcinoma

**DOI:** 10.1080/21655979.2021.1995574

**Published:** 2021-12-02

**Authors:** Zhenyu Yang, Bo Peng, Yongbo Pan, Yinmin Gu

**Affiliations:** a School of Biomedical Engineering (Suzhou), Division of Life Sciences and Medicine, University of Science and Technology of China, Hefei, China; b Cas Key Laboratory of Bio-medical Diagnostics, Suzhou Institute of Biomedical Engineering and Technology, Chinese Academy of Sciences, Suzhou, China; c Shanxi Academy of Advanced Research and Innovation, Taiyuan, China

**Keywords:** m^6^A, co-expression network, METTL14, biomarker, clear cell renal cell carcinoma

## Abstract

*N*^6^-methyladenosine (m^6^A) has been involved in diverse biological processes in cancer, but its function and clinical value in clear cell renal cell carcinoma (ccRCC) remain largely unknown. In this study, we found that 1453 m^6^A-modified differentially expressed genes (DEGs) of ccRCC were mainly enriched in cell cycle, PI3K-AKT, and p53 signaling pathways. Then we constructed a co-expression network of the 1453 m^6^A-modified DEGs and identified a most clinically relevant module, where NUF2, CDCA3, CKAP2L, KIF14, and ASPM were hub genes. NUF2, CDCA3, and KIF14 could combine with a major RNA m^6^A methyltransferase METTL14, serving as biomarkers for ccRCC. Real-time quantitative PCR assay confirmed that NUF2, CDCA3, and KIF14 were highly expressed in ccRCC cell lines and ccRCC tissues. Furthermore, these three genes were modified by m^6^A and negatively regulated by METTL14. This study revealed that NUF2, CDCA3, and KIF14 were m^6^A-modified biomarkers, representing a potential diagnostic, prognostic, and therapeutic target for ccRCC.

**Abbreviations:** m^6^A: *N*^6^-methyladenosine; ccRCC: clear cell renal cell carcinoma; DEGs: differentially expressed genes; NUF2: NUF2 component of NDC80 kinetochore complex; CDCA3: cell division cycle associated 3; CKAP2L: cytoskeleton associated protein 2 like; KIF14: kinesin family member 14; ASPM: assembly factor for spindle microtubules; METTL14: methyltransferase 14; OS: overall survival; FPKM: fragments per kilobase million; GEO: gene expression omnibus; TCGA: the Cancer Genome Atlas; RMA: robust multi-array average expression measure; WGCNA: weighted gene co-expression network analysis; GO: gene ontology; KEGG: kyoto encyclopedia of genes and genomes; ROC: receiver operating characteristic curve; AUC: area under the curve; RIP: RNA immunoprecipitation; qPCR: real-time quantitative PCR.

## Introduction

Renal cancer is the most commonly diagnosed cancer, with approximately 73,750 new cases and 14,830 death cases in the United States in 2020 [1]. Clear cell renal cell carcinoma (ccRCC) accounts for about 85% of all types of renal cancer [2]. Considerable progress has been made in diagnostic techniques, but 30% of ccRCC patients still suffer local invasion or distant metastasis at the initial diagnosis [3]. Molecular targeted therapy applied in the clinic did not make the situation of low overall survival (OS) of ccRCC patients better [4], thus, novel and effective biomarkers for ccRCC are urgently needed.

*N*^6^-methyladenosine (m^6^A) is an important RNA modification in mammals, linked to diverse effects in human cancers through the regulation of m^6^A-modified gene expression. For example, m^6^A-modified PDK4 regulates the glycolysis of cancer cells [5]. ALKBH5 enhances the expression of AURKB to promote renal cell carcinoma tumorigenesis in an m^6^A-dependent manner [6]. Thus, exploring the characteristics and function of m^6^A modification is crucial to monitoring and curing cancer. Although m^6^A regulators have been reported to be potential prognostic biomarkers for ccRCC [7,8], the prognostic value and clinical significance of m^6^A-modified genes in ccRCC remain unelucidated.

We hypothesized that m^6^A-modified genes can help monitor ccRCC and predict patient outcome. In this study, we analyzed ccRCC patients from the public cancer databases (TCGA and GEO) and performed experimental verification. We identified collective and hub m^6^A-modified genes related to ccRCC progression and demonstrated that the upregulated genes (NUF2, CDCA3, and KIF14) were modified by m^6^A and negatively regulated by METTL14 in ccRCC. Importantly, NUF2, CDCA3, and KIF14 could predict prognosis in patients with advanced ccRCC. Therefore, our work provides predictive biomarkers for for ccRCC.

## Materials and methods

### Public data collection

The TCGA-mRNA FPKM data of ccRCC patients and matched clinical information, including 535 tumor tissues and 72 normal tissues, were downloaded from UCSC Xena (http://xenabrowser.net/). Gene expression profiling data and related clinical information, including 27 ccRCC tissues and 27 adjacent normal tissues, were downloaded from the GEO database, accession No.GSE66272 [9]. The high confidence level of m^6^A-modified human protein-coding genes were collected from the m6Avar database (http://m6avar.renlab.org/) [10].

### Data processing and DEGs screening

The ‘affy’ R package and RMA method were utilized for background correction and log_2_ transformation of raw data [11]. The ‘limma’ R package [12] was applied to screen DEGs between normal adjacent tissues and tumor tissues by setting *p*-value < 0.05 and | log_2_ Fold change | > 0.5.

### Weighted co-expression network construction

The weighted gene co-expression network analysis was performed using the ‘WGCNA’ R package [13]. The suitable soft threshold power *β* was selected to achieve a scale-free network. We calculated the adjacencies between candidate m^6^A-modified DEGs and then turned the adjacency matrix into a topological overlap matrix (TOM) [14]. According to the dissimilarity (1-TOM) measurement and the minimum size of 30 genes for gene dendrogram, average linkage and hierarchical clustering were conducted. Highly similar modules were merged according to the height cutoff of 0.25.

### Analysis of clinically relevant modules and hub m^6^A-modified genes

The module eigengene (ME) could represent the gene expression profiles from a given module [15]. The correlations between MEs and clinical traits were calculated to identify the clinically relevant modules. Gene significance (the correlation between gene expression and clinical trait, GS) and module membership (the correlation between MEs and gene expression, MM) of all genes in the most clinically relevant module was calculated [16]. GS > 0.6 and MM > 0.8 were defined to screen hub m^6^A-modified genes in the target module. Besides, genes with a minimum weight greater than 0.8 were selected to build a PPI (protein-protein interaction) network, and Cytoscape plotted the network [17].

### Functional annotation analysis

GO functional enrichment and KEGG pathway enrichment were applied with the ‘clusterProfiler’ R package [18] to reveal the potential biological mechanisms of m^6^A-modified DEGs involved in correlative clinical features. *p*-value < 0.05 was set as the threshold.

### Survival analysis and ROC analysis

Kaplan-Meier analysis was conducted to evaluate the OS of ccRCC patients grouped by target genes [19]. ROC curves and AUC values of multiple genes were performed by the ‘pROC’ R package [20].

### qPCR

Total RNA was extracted using TRIzol reagent according to the manufacture’s instructions. The relative expression of target genes was compared by the relative quantification equation (RQ = 2 ^−ΔΔCt^) [21]. The primers were shown in Supplementary Table 1. The information of cell lines was shown in Supplementary Table 2. The patients’ clinicopathologic data was shown in Supplementary Table 3.

### m^6^A RIP qPCR

300 μg of total RNA was sheared to 100–150 nt in length by fragmentation reagents, then incubated with anti-m^6^A antibody (Synaptic Systems, 202,003)-conjugated beads in 1 mL 1× immunoprecipitation buffer at 4°C overnight [22]. m^6^A-methylated RNA was immune-precipitated with beads, then eluted and recovered with RNeasy kit (QIAGEN). The product RNA was analyzed by qPCR. The primers were shown in Supplementary Table 1.

### siRNA transfection

For analysis of the NUF2, CDCA3, and KIF14 levels in 769-P cells after transient depletion of endogenous METTL14 with RNAi, 769-P cells were transfected with 50 nM pool siRNA against METTL14 using jetPRIME (Polyplus Transfection, Cat.#114-07). As control, the cells were transfected with 50 nM of the si-control RNA. The transfection media was replaced 12 hour post-transfection with fresh growth media. siRNA #1, GGAUGAAGGAGAGACAGAUTT; siRNA #2, GCAGCACCUCGAUCAUUUATT; siRNA #3, CCUGGGAAGACUAAGACUUTT.

### Statistical analysis

The Student’s *t*-test was used to assess the significant differences between the two groups. Pearson correlation of two target genes was calculated and plotted using ‘corrplot’ and ‘ggstatsplot’ R package [23]. All statistical results with *p* < 0.05 were considered statistically significant.

## Results

The purpose of this study was to identify m^6^A-modified genes and provide potential biomarkers for ccRCC. We systematically analyzed the m^6^A-modified genes of ccRCC and finally obtained 1453 candidate genes. Then these genes were used to build the co-expression modules. A most clinically relevant module was identified, where NUF2, CDCA3, and KIF14 were hub genes and negatively correlated with METTL14. Moreover, NUF2, CDCA3, and KIF14 were confirmed to be m^6^A-modified biomarkers and negatively regulated by METTL14. Our results suggested that m^6^A-modified genes (NUF2, CDCA3, and KIF14) were potential prognostic and therapeutic targets for ccRCC.

### Identification of m^6^A-modified DEGs in ccRCC

**Figure 1.**
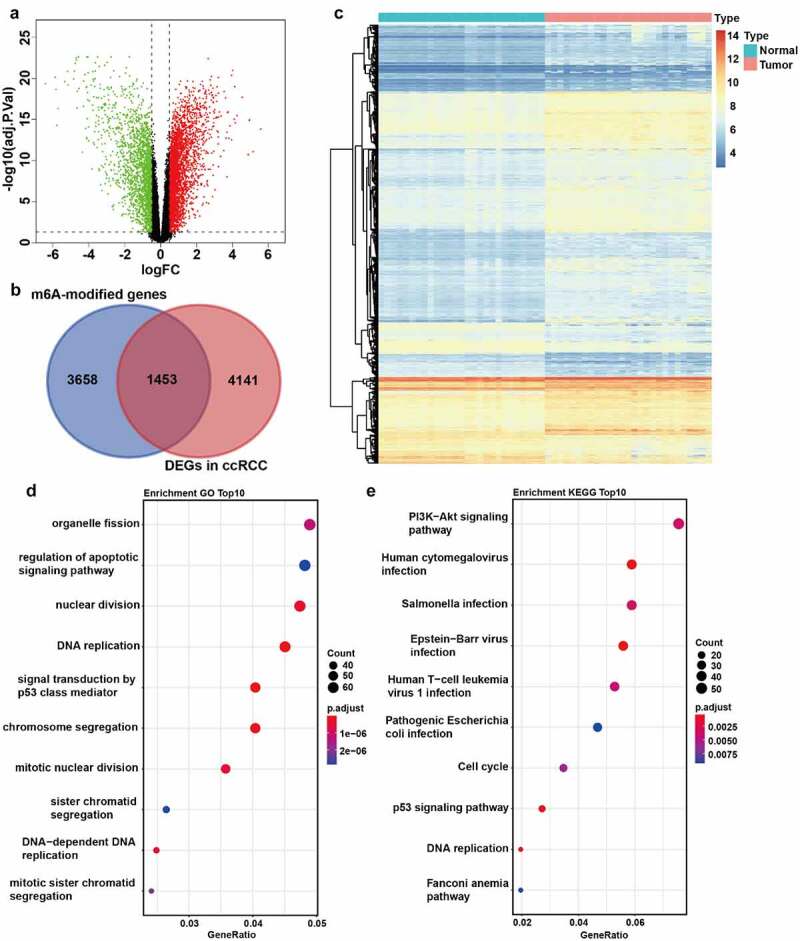
**Identification of m^6^A-modified DEGs between ccRCC tissues and normal tissues. A** The volcano plot visualizes the DEGs in GSE66272. Green dots represent downregulated genes and red dots represent upregulated genes. FC, Fold Change. Adj.P.Val, adjust P value. **B** The Venn plot of m^6^A-modified DEGs in ccRCC. **C** The mRNA levels of m^6^A-modified DEGs in both ccRCC tissues and adjacent normal tissues from GSE66272. **D-E** GO (d) and KEGG (e) pathway analysis of m^6^A-modified DEGs in ccRCC

The m^6^A-modified genes involved in the progression of ccRCC were identified using the dataset GSE66272 firstly. A total of 5594 DEGs between tumor and normal tissues were screened, including 3441 upregulated genes and 2153 downregulated genes (Figure 1a, Supplementary File 1). Additionally, a total of 5111 m^6^A-modified (high confidence level) human protein-coding genes were collected from the m6Avar database (Supplementary File 2). An integrated analysis was performed on the DEGs and m^6^A-modified genes, which identified 1453 candidate genes as m^6^A-modified DEGs of ccRCC (Figure 1b-c). To evaluate how m^6^A-modified DEGs affect the progression of ccRCC patients, functional enrichment analysis was performed, showing that these genes were enriched in cell division, DNA replication, cell cycle, PI3K-AKT, and p53 pathways, which were related to cancer (Figure 1d-e). These signaling pathways were the biological mechanisms of m^6^A modification to promote ccRCC progression possibly.

### Detection of m^6^A-modified DEGs by WGCNA

**Figure 2.**
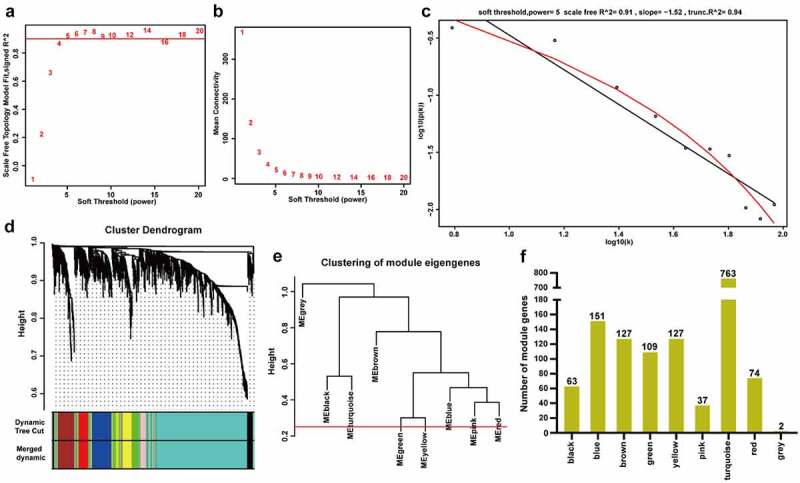
**Construction of weighted co-expression modules. A** The scale-free topology model fits the index for various soft threshold powers. **B** The mean connectivities for various soft threshold powers. **C** Checking the scale-free R^2^ when the power of *β* = 5. **D** The cluster dendrogram of the m^6^A-modified DEGs in GSE66272. **E** The clustering of module eigengenes (MEs). **F** The number of genes in different modules

To identify the hub m^6^A-modified genes involved in ccRCC progression, we constructed a co-expression network of the 1453 m^6^A-modified DEGs through WGCNA. *β* = 5 was chosen to achieve a scale-free network (scale-free R^2^ = 0.91) (Figure 2a-c). MEDissThres = 0.25 was set to merge highly similar modules, and nine co-expression modules were obtained (Figure 2d-e). The number of genes in each co-expression module was shown in figure 2f.

### Analysis of clinically relevant modules and hub m^6^A-modified genes

**Figure 3.**
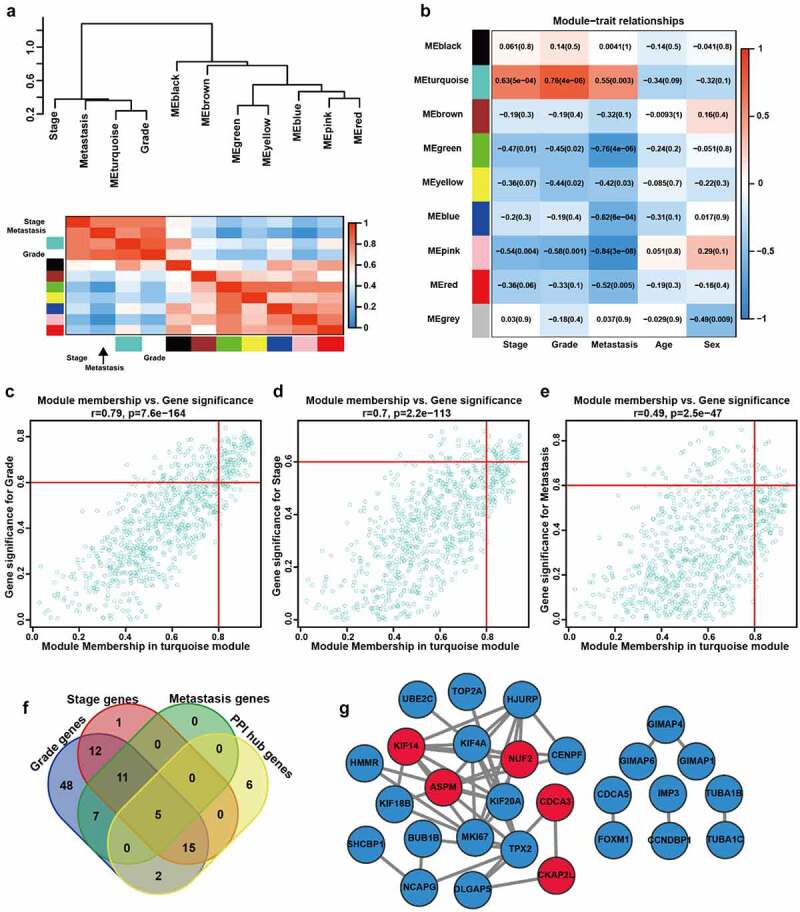
**Analysis of clinically relevant modules and hub m^6^A-modified genes. A** The modules yielded in the clustering analysis are summarized by ME dendrogram and network heatmap. **B** The heatmap of the correlations between clinical traits and MEs. **C-E** Scatterplot visualizes the ModuleMembership and GeneSignificance of genes in the turquoise module based on grade (c), stage (d) and metastasis (e) of GSE66272. r, Pearson correlation coefficient. p, P value. **F** The Venn plot of the most clinically significant genes. **G** Protein-protein network of genes with weight > 0.8. Red represents the genes that belong to both turquoise module and PPI network, blue represents the genes that only belong to PPI network

We calculated the module eigengene (ME) of these modules and performed clustering analysis according to their correlation. The results showed that the eight modules (the gray module was not included) were separated into two clusters, consistent with the result of the eigengene network heatmap (Figure 3a). Compared with other modules, the turquoise module was remarkly correlated with tumor grade, stage, and metastasis of ccRCC patients (Figure 3b). Therefore, the turquoise module was chosen for subsequent analysis. We firstly identified module hub genes (Figure 3c-e, Supplementary File 3), then constructed PPI network of genes with a minimum weight greater than 0.8 (Supplementary File 4). Five genes (NUF2, CDCA3, CKAP2L, KIF14, and ASPM) were included in both module hub genes and PPI network genes, identified as hub m^6^A-modified genes of ccRCC (figure 3f-g).

### Hub m^6^A-modified genes of ccRCC are associated with poor outcomes

TCGA dataset was further analyzed to confirm clinic significance of NUF2, CDCA3, CKAP2L, KIF14, and ASPM in ccRCC patients. The results showed that the expression levels of these five genes in ccRCC tissues were significantly higher than adjacent normal tissues (Supplementary Figure 1a), significantly associated with poor outcomes, including grade, pathologic stage, and metastasis of ccRCC patients, respectively (Supplementary Figure 1b-d).

### NUF2, CDCA3, and KIF14 are modified by m^6^A and negatively regulated by METTL14

**Figure 4.**
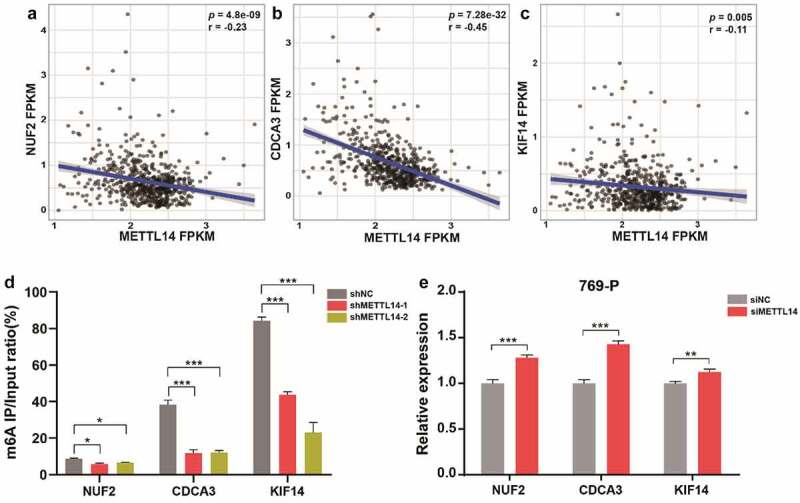
**METTL14 regulates the m^6^A and expression levels of NUF2, CDCA3, and KIF14. A-C** Pearson correlation analysis of METTL14 and NUF2 (a), CDCA3 (b), KIF14 (c) in TCGA dataset. r, Pearson correlation coefficient. p, P value. **D** The m^6^A RIP qPCR assay shows the enrichment levels of m^6^A modification in NUF2, CDCA3, and KIF14 transcripts. **E** The relative expression of NUF2, CDCA3, and KIF14 after METTL14 knockdown in 769-P cells

It was reported that decreased expression of a major RNA m^6^A methyltransferase METTL14 could predict a poor prognosis of ccRCC [24]. Therefore, we investigated the associations between METTL14 and these five m^6^A-modified genes, the results showed that NUF2, CDCA3, and KIF14 were negatively correlated (*p* < 0.01) with METTL14 (Figure 4a-c). To further validate this finding, we performed m^6^A RIP qPCR assay and confimed that METTL14 knockdown remarkly reduced the m^6^A methylation levels of NUF2, CDCA3, and KIF14, suggesting that these three genes were modified by m^6^A (Figure 4d). Furthermore, qPCR assay showed that METTL14 knockdown significantly upregulated the expression of NUF2, CDCA3, and KIF14 in 769-P cells (Figure 4e), indicating that METTL14 negatively regulates NUF2, CDCA3, and KIF14 through m^6^A modification probably.

### NUF2, CDCA3, and KIF14 have a prognostic value in ccRCC

**Figure 5.**
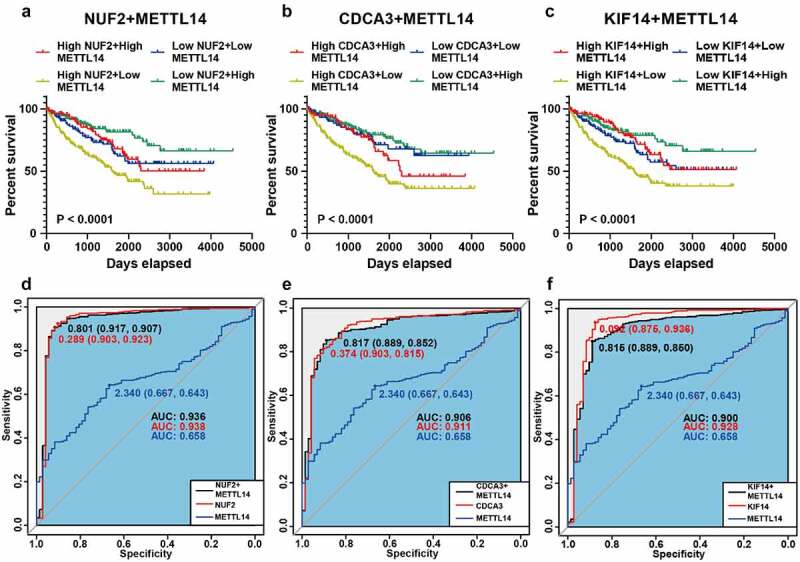
**Survival and ROC analysis of METTL14 and NUF2, CDCA3, and KIF14. A-C** Kaplan-Meier analysis of overall survival based on METTL14 and NUF2 (a), CDCA3 (b), KIF14 (c) in TCGA ccRCC patients. **D-F** Combined ROC curves of METTL14 and NUF2 (d), CDCA3 (e), and KIF14 (f) according to the expression levels

To further assess the clinical values of NUF2, CDCA3, and KIF14 in ccRCC patients, we performed combined survival analysis and ROC analysis of METTL14 and these three m^6^A-modified genes, respectively. Kaplan-Meier analysis revealed that patients with higher expression of NUF2 and lower expression of METTL14 tended to have a poorer prognosis (Figure 5a). Moreover, ROC analysis showed that combining METTL14 with NUF2 could effectively distinguish ccRCC tissues from adjacent normal tissues (Figure 5d). The results of CDCA3 and KIF14 were the same as those of NUF2 (Figure 5b-c and e-f). Moreover, combining METTL14 with m^6^A-modified genes (NUF2, CDCA3, and KIF14) could have better prognostic and diagnostic potential than METTL14 alone. Thus, NUF2, CDCA3, and KIF14 can serve as biomarkers for ccRCC in the combination of METTL14.

### ccRCC tissue and cell line validation for NUF2, CDCA3, and KIF14

**Figure 6.**
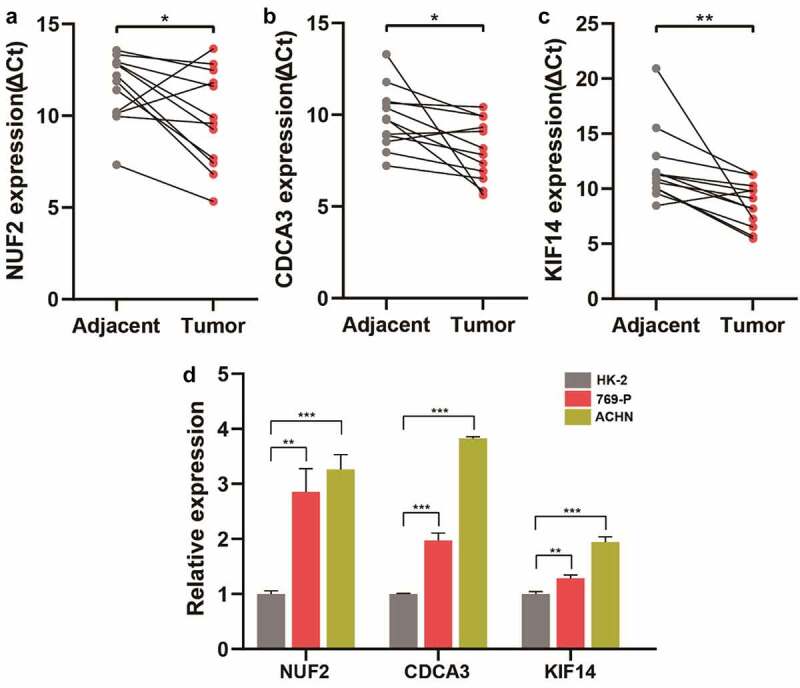
**Validation of NUF2, CDCA3, and KIF14 in ccRCC tissues and cell lines. A-C** Detection of NUF2 (a), CDCA3 (b), and KIF14 (c) expression levels in 12 paired ccRCC and normal kidney tissues by qPCR. The expression levels of these three genes were normalized to the reference gene GAPDH. ΔCt, delta threshold cycle. **D** Detection of NUF2, CDCA3, and KIF14 expression levels in kidney cell lines by qPCR

To further investigate the clinical significance of NUF2, CDCA3, and KIF14, a cohort containing 12 pairs of ccRCC and matched adjacent tissues were analyzed. qPCR assay showed that NUF2, CDCA3, and KIF14 expression significantly increased in ccRCC tissues versus adjacent normal tissues (Figure 6a-c). Additionally, compared with the immortalized human HK-2 tubular epithelial cell line, NUF2, CDCA3, and KIF14 were significantly higher expressed in ccRCC cell lines (769-P and ACHN) (Figure 6d), suggesting that NUF2, CDCA3, and KIF14 are over-expressed genes and might be biomarkers for ccRCC.

## Discussion

ccRCC is a heterogeneous tumor driven by oncogene activation and tumor suppressor gene inactivation [25]. There are few treatment strategies for advanced ccRCC patients due to the lack of effective therapeutic targets and diagnostic markers. Although some single molecules were proposed to serve as prognostic biomarkers for ccRCC, lacking ample credibility [26]. Therefore, identifying novel biomarkers is necessary. Recently, aberrant m^6^A abundance in specific genes has been reported in cancers, suggested to have associations with cancer progression and clinical outcome [27,28], indicating that m^6^A abundance could monitor cancers. Here, the study analyzed the m^6^A-modified genes with prognostic values in ccRCC and revealed that NUF2, CDCA3, and KIF14 were strongly associated with ccRCC progression, and could serve as biomarkers for ccRCC in combination with METTL14.

METTL14 has been reported to be a tumor suppressor gene in many cancers [29]. Low expression of METTL14 in hepatocellular carcinoma was related to poor prognosis and tumor metastasis through regulating pri-miR126 [30]. METTL14 knockdown had a positive impact on glioblastoma stem cell growth by decreasing the expression of ADAM19 in an m^6^A-dependent manner [31]. Although METTL14 was reported to be an independent prognostic factor for ccRCC [24], combining METTL14 with m^6^A-modified genes could have better diagnostic and prognostic potential than using METTL14 alone. However, the function and molecular mechanism of METTL14 affecting ccRCC remain enigmatic.

Besides, numerous genes regulated by m^6^A have not yet been revealed in ccRCC. We identified that NUF2, CDCA3, and KIF14 were hub genes in ccRCC progression and modified by m^6^A. NUF2 is a component of the kinetochore protein complex, crucial in chromosome segregation. Inhibition of NUF2 can arrest stable kinetochore-microtubule attachment and induce mitotic cell death [32]. NUF2 is an oncogene regulated by CircFOXK2 in pancreatic ductal adenocarcinoma [33]. CDCA3 is an F-box-like protein required for entry into mitosis [34]. CDCA3 promotes cell proliferation by activating the NF-κB/cyclin D1 axis in colon cancer [35], which is probably a potential prognostic biomarker in gastric cancer [36]. KIF14 belongs to the kinesin-3 superfamily of microtubule motor proteins. KIF14 can promote tumor progression and serve as an independent biomarker in human gastric cancer [37]. KIF14 can also promote AKT phosphorylation and contribute to chemoresistance in triple-negative breast cancer therapy [38]. Several studies have revealed the oncogenic role of NUF2, CDCA3, and KIF14 in tumor, but the mechanism has not been elucidated. This paper suggests that the expression of NUF2, CDCA3, and KIF14 are negatively regulated by METTL14, which may be mediated by m^6^A modification. However, the mechanism of METTL14 in regulating them needs to be studied further.

Additionally, several limitations in this study should be acknowledged. First, most data in this study were acquired from the public database. Second, further studies, including molecular mechanism and phenotypic experiments, are needed to be conducted.

## Conclusion

In conclusion, we revealed that NUF2, CDCA3, and KIF14 are novel m^6^A-modified biomarkers for ccRCC, which might provide a new perspective for clinical diagnose and treatment. More importantly, our study sheds new light on the research of m^6^A modification in ccRCC progression.

## Supplementary Material

Supplemental Material

## Highlights

1. m^6^A methylation may promote ccRCC progression through cell cycle, PI3K-AKT, and p53 signaling pathways.
2. METTL14 negatively regulates the mRNA levels of NUF2, CDCA3, and KIF14 probably through m^6^A.
3. Combining METTL14 with m^6^A-modified genes (NUF2, CDCA3, and KIF14) can help diagnose and prognose ccRCC patients.
