# Supplementary material for: Analysis and verification of N6-methyladenosine-modified genes as novel biomarkers for clear cell renal cell carcinoma
Source: Bioengineered. 2021 Dec 2;12(2):9473–83. doi: 10.1080/21655979.2021.1995574 (PMC8810125; doi:10.1080/21655979.2021.1995574)
Supplement: Supplemental Material [file KBIE_A_1995574_SM9622.zip › supplementary/Supplementary Fig legend.docx]

**Supplementary Fig. 1 The associations between hub m^6^A-modified genes and clinical traits in ccRCC. A** The relative mRNA levels of NUF2, CDCA3, CKAP2L, KIF14, and ASPM in tumor tissues and normal adjacent tissues across TCGA ccRCC cohort. **B-D** The relative mRNA levels of NUF2, CDCA3, CKAP2L, KIF14, and ASPM grouped by grade (G1+2 versus G3+4), stage (Stage I+II versus Stage III+IV), and metastasis (M0 versus M1).
