# Supplementary material for: Analysis and verification of N6-methyladenosine-modified genes as novel biomarkers for clear cell renal cell carcinoma
Source: Bioengineered. 2021 Dec 2;12(2):9473–83. doi: 10.1080/21655979.2021.1995574 (PMC8810125; doi:10.1080/21655979.2021.1995574)
Supplement: Supplemental Material [file KBIE_A_1995574_SM9622.zip › supplementary/YangSupplementaryTable.docx]

**Supplementary Table 1. List of primers used to detect hub m^6^A-modified genes**

| **Gene name** | **Application** | **Forward Primer (5’-3’)** | **Reverse Primer (5’-3’)** |
| --- | --- | --- | --- |
| NUF2 | qPCR | TAGGAGGCGGCAAGTTTGAA | CTGAGCACGACGAAAACACC |
| CDCA3 | qPCR | TGCGCAATAGATGGAAACCA | CCATGCTCGTCCTCCAGTTT |
| KIF14 | qPCR | TTCAAATTGCGGCCTTCTGGG | CTGAGGAACTGAATGGGGGAC |
| NUF2 | m6A RIP qPCR | ATGCTGCTGAAAGGGAGAAACT | ACATCTTCCTCTTCAGTTCAGC |
| CDCA3 | m6A RIP qPCR | AGGTGGTGACGAAAAAGATATGGCGAG | CTACAGTCTACAGTCTATATTCAAATTTCACC |
| KIF14 | m6A RIP qPCR | TGCCCCCAGTAGAGCAAATG | ACAAGTTTCCTGTTCCCAACAC |

**Supplementary Table 2. Cell lines and media**

| **Cell line** | **Media** |
| --- | --- |
| HK-2 | DMEM/F-12 |
| 786-O | RPMI-1640 |
| 769-P | RPMI-1640 |
| ACHN | RPMI-1640 |

**Supplementary Table 3. Clinicopathologic characteristics of ccRCC patients from the General Hospital of the People's Liberation Army cohort**

| **Characteristics** | **Number of cases** |
| --- | --- |
| **Gender** |  |
| Male | 11 |
| Female | 1 |
| **Age (years)** |  |
| ≥ 60 | 3 |
| ＜ 60 | 9 |
| **WHO/ISUP** |  |
| I+II | 4 |
| III+IV | 1 |
| Data missing | 7 |
| **Furman grade** |  |
| I+II | 5 |
| III+IV | 0 |
| Data missing | 7 |
| **T classification** |  |
| T1 | 10 |
| T2 | 0 |
| T3 | 2 |
| **N classification** |  |
| N0 | 11 |
| N1 | 1 |
